# Identification of microRNAs expressed in two mosquito vectors, *Aedes albopictus *and *Culex quinquefasciatus*

**DOI:** 10.1186/1471-2164-11-119

**Published:** 2010-02-18

**Authors:** Rebecca L Skalsky, Dana L Vanlandingham, Frank Scholle, Stephen Higgs, Bryan R Cullen

**Affiliations:** 1 Department of Molecular Genetics and Microbiology and Center for Virology, Duke University Medical Center, Durham, NC 27710, USA; 2 Department of Microbiology, North Carolina State University, Raleigh, NC 27695, USA; 3 Department of Pathology and Department of Microbiology and Immunology, University of Texas Medical Branch, Galveston, TX, USA

## Abstract

**Background:**

MicroRNAs (miRNAs) are small non-coding RNAs that post-transcriptionally regulate gene expression in a variety of organisms, including insects, vertebrates, and plants. miRNAs play important roles in cell development and differentiation as well as in the cellular response to stress and infection. To date, there are limited reports of miRNA identification in mosquitoes, insects that act as essential vectors for the transmission of many human pathogens, including flaviviruses. West Nile virus (WNV) and dengue virus, members of the *Flaviviridae *family, are primarily transmitted by *Aedes *and *Culex *mosquitoes. Using high-throughput deep sequencing, we examined the miRNA repertoire in *Ae. albopictus *cells and *Cx. quinquefasciatus *mosquitoes.

**Results:**

We identified a total of 65 miRNAs in the *Ae. albopictus *C7/10 cell line and 77 miRNAs in *Cx. quinquefasciatus *mosquitoes, the majority of which are conserved in other insects such as *Drosophila melanogaster *and *Anopheles gambiae*. The most highly expressed miRNA in both mosquito species was miR-184, a miRNA conserved from insects to vertebrates. Several previously reported *Anopheles *miRNAs, including miR-1890 and miR-1891, were also found in *Culex *and *Aedes*, and appear to be restricted to mosquitoes. We identified seven novel miRNAs, arising from nine different precursors, in C7/10 cells and *Cx. quinquefasciatus *mosquitoes, two of which have predicted orthologs in *An. gambiae*. Several of these novel miRNAs reside within a ~350 nt long cluster present in both *Aedes *and *Culex*. miRNA expression was confirmed by primer extension analysis. To determine whether flavivirus infection affects miRNA expression, we infected female *Culex *mosquitoes with WNV. Two miRNAs, miR-92 and miR-989, showed significant changes in expression levels following WNV infection.

**Conclusions:**

*Aedes *and *Culex *mosquitoes are important flavivirus vectors. Recent advances in both mosquito genomics and high-throughput sequencing technologies enabled us to interrogate the miRNA profile in these two species. Here, we provide evidence for over 60 conserved and seven novel mosquito miRNAs, expanding upon our current understanding of insect miRNAs. Undoubtedly, some of the miRNAs identified will have roles not only in mosquito development, but also in mediating viral infection in the mosquito host.

## Background

*Culex *and *Aedes *mosquitoes are members of the Culicinae subfamily that vector positive-sense RNA viruses from the family *Flaviviridae*. Many flaviviruses, such as West Nile virus (WNV), dengue virus (DENV), and yellow fever virus (YFV), are highly pathogenic in humans and pose an important health problem worldwide [1]. Each year, an estimated 50 million human cases of dengue fever occur due to infection with DENV. Since the introduction of WNV to the United States in 1999, over 28,000 cases have been reported to the CDC, with approximately 3,000 cases annually http://CDC.gov. *Culex *mosquitoes are primarily responsible for the transmission of WNV to humans (reviewed in [2]), although WNV has also been isolated from *Aedes albopictus *in the eastern United States (reviewed in [3]). Virus transmission from *Cx. quinquefasciatus *occurs as early as five days following an infectious blood meal [4], and virus can persist as long as four weeks in the midguts and salivary glands of infected mosquitoes [5,6].

Both *Culex *and *Aedes *mosquitoes are prevalent in tropical and subtropical regions around the world. Recently, *Ae. albopictus *has emerged as a major vector for Chikungunya virus, an alphavirus, in regions bordering the western Indian Ocean [7,8]. *Ae. albopictus *is also considered a secondary vector for dengue virus serotypes 1-4 (DENV1-4) and YFV, which are predominantly transmitted to humans by a mosquito from the same genus, *Ae. aegypti. Ae. albopictus *can potentially vector at least 22 known arboviruses (reviewed in [3]).

Of the over 3,000 mosquito species worldwide, microRNAs (miRNAs) have so far only been described in two species of African malaria mosquitoes, *Anopheles gambiae *and *Anopheles stephensi*, using direct cloning and computational methods. Over 55 miRNAs have been described for *Anopheles *mosquitoes, at least 49 of which have orthologs in *Drosophila melanogaster *and other insects [9-12]. The functions of these miRNAs in mosquitoes, and the identities of their mRNA targets, are not yet known.

miRNAs are a class of small, non-coding RNAs, from 19-24 nt in length, that post-transcriptionally regulate gene expression by binding to complementary regions in, primarily, the 3' untranslated region (3' UTR) of target messenger RNAs. First identified in *Caenorhabditis elegans*, miRNAs have now been found in a wide variety of organisms including insects, vertebrates, and plants [13-15]. Over 10,800 miRNAs are currently annotated in miRBase, many of which are conserved from worms to flies to humans [9]. In humans, miRNAs are predicted to regulate as much as one-third of all mRNAs [16], and thus, represent an important component in managing biological processes.

Much of what we understand about insect miRNAs comes from studies in the fruit fly *D. melanogaster*. *D. melanogaster *miRNAs were originally identified via direct cloning of small RNA molecules and many of these miRNAs exhibited significant sequence conservation with miRNAs expressed in *C. elegans *[17]. At present, 147 different miRNAs have been annotated for *D. melanogaster*, the majority of which have orthologous sequences in other winged insects [9]. With the identification of new miRNAs in a number of organisms, evolutionary sequence conservation has become a hallmark of miRNA biology [12,15,18,19].

Differential miRNA expression throughout the various stages of the *Drosophila *life cycle has revealed a role for miRNAs in important cellular processes such as apoptosis, cell division, and differentiation [20-22]. Additionally, miRNA expression profiles change in response to stress, inflammation, and infection [11,19]. For example, in *Anopheles *mosquitoes, expression levels of four miRNAs are altered during the response to *Plasmodium *infection [11].

The process of miRNA biogenesis is conserved, initiating with the cleavage of long, endogenous nuclear primary miRNA transcripts, ranging from hundreds to thousands of nucleotides in length, into pre-miRNAs [23,24]. Two proteins are required for this processing in insects, the RNAse III enzyme Drosha and its binding partner Pasha, which together excise the ~60 nt pre-miRNA hairpin from the pri-miRNA [25]. The pre-miRNA is then exported to the cytoplasm and processed by a second RNAse III enzyme called Dicer-1 to yield the ~22 bp miRNA:miRNA* duplex intermediate [13]. Mature miRNAs are selectively loaded into the multi-component RNA-induced silencing complex (RISC) which contains members of the Argonaute family (Ago). In *Drosophila*, strand selection has been shown to depend on the intrinsic structure of the miRNA:miRNA* duplex, which facilitates sorting into either Ago1- or Ago2-containing RISCs [26,27]. Recent comparative genomics studies have shown that the *Anopheles, Aedes*, and *Culex *mosquito genomes all encode orthologs of key proteins involved in the miRNA, as well as small interfering RNA (siRNA) and piwi RNA (piRNA), regulatory pathways [28]. Mature miRNAs are used as guide RNAs to direct RISC to complementary regions of mRNAs, resulting in the inhibition of translation and/or target mRNA degradation. Important for this targeting are nucleotides 2-8 from the 5' end of the mature miRNA, known as the "seed" [29,30]. Many studies have shown that miRNAs can target 3'UTRs of mRNAs [31,32]; however, recent studies have also revealed functional target sites within the ORFs of mRNAs [33,34].

Recent advances in mosquito genomics, such as the sequencing of the genomes of three mosquito species, *Ae. aegypti, Cx. quinquefasciatus*, and *An. gambiae *[35], together with technological advances in small RNA cloning methods, enabled us to interrogate the miRNA repertoire in two flavivirus mosquito vectors. In this study, we used deep sequencing to identify over 60 conserved and several novel miRNAs in *Cx. quinquefasciatus *mosquitoes and an *Ae. albopictus *cell line, C7/10, commonly used for *in vitro *flavivirus studies. We additionally investigated the effects of flavivirus infection on miRNA expression and found that miR-92 and miR-989 are significantly changed in response to WNV infection.

## Results and Discussion

### Deep sequencing of small RNAs

**Figure 1:**
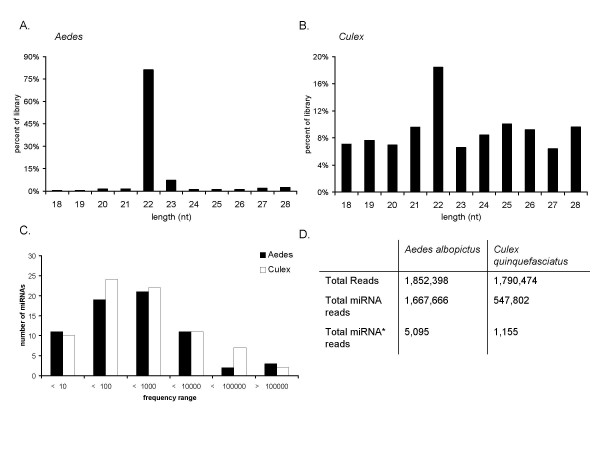
**Deep sequencing of small RNA populations in *Ae. albopictus *C7/10 cells and *Cx. quinquefasciatus *mosquitoes**. Size distributions of small RNA libraries (18-28 nt) from A) C7/10 cells (*Aedes*) and B) *Cx. quinquefasciatus *(*Culex*). C) Frequencies of read counts for individual, conserved miRNAs present in C7/10 cells and *Culex *mosquitoes. Expression levels, based on read counts, of individual miRNAs are separated into several ranges and span over 5 orders of magnitude. D) Breakdown of the total number of reads obtained for each library. The number of reads mapping to miRNA and miRNA* strands is reported.

To identify miRNAs in *Culex *and *Aedes *mosquitoes, we isolated small RNAs (18-28 nt) from C7/10 *Ae. albopictus *cells and blood-fed, female *Cx. quinquefasciatus *mosquitoes. Small RNA libraries were subjected to Illumina-based high-throughput sequencing. After filtering for linker sequences, and removing ambiguous reads, a total of 1,852,398 reads for *Ae. albopictus *cells and 1,790,474 reads for *Cx. quinquefasciatus *mosquitoes, representing 41,056 and 281,918 non-redundant sequences, respectively, were obtained (Figure 1D). >90% of final reads for *Ae. albopictus *and >50% of reads for *Cx. quinquefasciatus *exhibited the predominantly ~22 nt size expected for insect miRNAs (Figure 1A, B).

### Most mosquito miRNAs are orthologs of known insect miRNAs

We aligned sequencing reads to known miRNAs and miRNA* strands present in miRBase v14. 1,541,048 reads from the *Ae. albopictus *library corresponded to 53 distinct pre-miRNAs (61 miRNAs) (Table 1). For the *Cx. quinquefasciatus *library, 382,878 reads aligned to sequences present in miRBase, representing 69 distinct pre-miRNAs (74 miRNAs) (Table 2). For each miRNA, the sequence with the greatest number of reads was annotated and named according to the most similar match in miRBase [9]. In addition to mature miRNAs, we identified a number of miRNA* strands (Figure 1D, Tables 1, 2), which accounted for < 0.2% of the 20-24 nt population. 21 and 33 distinct miRNA* strands were identified in *Ae. albopictus *and *Cx. quinquefasciatus *respectively, and are orthologous to miRNA* strands in other winged insects (Tables 1, 2).

**Table 1:** miRNAs identified in *Ae. albopictus *C7/10 cells and predicted in *Ae. aegypti*.

C710	# miRNA	# miRNA*	Sequence	Length	aga	ame	dme	Ae. aegypti	Start	End	Strand
184	1487481	0	UGGACGGAGAACUGAUAAGGGC	22	Y	Y	Y	1.496	143378	143399	Minus
275	23841	78	UCAGGUACCUGAAGUAGCGC	20	Y	Y	Y	1.24	486591	486610	Plus
277	4453	7	UAAAUGCACUAUCUGGUACGAC	22	Y	Y	Y	1.265	508860	508881	Plus
9	4085	602	UCUUUGGUAUUCUAGCUGUAGA	22	Y	Y	Y	1.785	186231	186252	Plus
8-3p	3002	-	UAAUACUGUCAGGUAAAGAUGUC	23	Y	Y	Y	1.411	876091	876113	Plus
252.1	1608	13	UAAGUACUAGUGCCGCAGGAG	21	Y	Y	Y	1.56	1580060	1580080	Minus
bantam-5p	1384	-	CCGGUUUUCAUUUUCGAUCUGAC	23	Y	Y	Y	1.49	157893	157915	Minus
71	1246	17	AGAAAGACAUGGGUAGUGAGAU	22	?	Y	?	1.268	889428	889449	Minus
8-5p	1244	-	CAUCUUACCGGGCAGCAUUAGA	22	Y	Y	Y	1.411	876052	876073	Plus
276-1	1209	4	UAGGAACUUCAUACCGUGCUC	21	Y	Y	Y	1.5	2769510	2769530	Minus
276-2	-	-	UAGGAACUUCAUACCGUGCUC	21	Y	Y	Y	1.134	39026	39046	Plus
317-1	1118	0	UGAACACAGCUGGUGGUAUCU	21	Y	Y	Y	1.265	429503	429523	Plus
317-2	-	-	UGAACACAGCUGGUGGUAUCU	21	Y	Y	Y	1.153	2154717	2154737	Minus
283	947	0	CAAUAUCAGCUGGUAAUUCUGGGC	24	Y	Y	Y	1.68	2729393	2729416	Minus
252.2	888	-	CUAAGUACUAGUGCCGCAGGAG	22	Y	Y	Y	1.56	1580060	1580081	Minus
let-7	650	0	UGAGGUAGUUGGUUGUAUAGU	21	Y	Y	Y	1.43	1156331	1156351	Plus
2	708	1	UAUCACAGCCAGCUUUGAAGAGC	23	Y	Y	Y	1.268	888597	888619	Minus
998	561	0	UAGCACCAUGAGAUUCAGC	19	?	?	Y	1.744	322338	322356	Plus
92b	530	0	AAUUGCACUUGUCCCGGCCUG	21	Y	Y	Y	1.116	1319201	1319221	Plus
1889-3p	478	-	CACGUUACAGAUUGGGGUUUCC	22	Y	?	?	1.68	2720796	2720817	Minus
bantam-3p	475	-	UGAGAUCAUUUUGAAAGCUGAU	22	Y	Y	Y	1.49	1579555	1579576	Minus
306	454	81	UCAGGUACUGAGUGACUCUCAG	22	Y	?	Y	1.785	213078	213099	Plus
281	398	1	AAGAGAGCUAUCCGUCGAC	19	Y	Y	Y	1.957	134462	134480	Plus
1889-5p	378	-	UAAUCUCAAAUUGUAACAGUGG	22	Y	?	?	1.68	2720896	2720917	Minus
980	309	6	UAGCUGCCUAGUGAAGGGC	19	?	?	Y	1.23	1043069	1043087	Plus
278	286	53	ACGGACGAUAGUCUUCAGCGGCC	23	Y	Y	Y	1.16	3596269	3596291	Plus
989	242	0	UGUGAUGUGACGUAGUGGUAC	21	Y	?	Y	1.115	804187	804207	Minus
14	247	0	UCAGUCUUUUUCUCUCUCCUAU	22	Y	Y	Y	1.249	1089019	1089040	Minus
11	222	31	CAUCACAGUCUGAGUUCUUGCU	22	Y	?	Y	1.744	322062	322083	Plus
190	210	0	AGAUAUGUUUGAUAUUCUUGGUUG	24	Y	Y	Y	1.195	82254	82277	Minus
1	169	0	UGGAAUGUAAAGAAGUAUGGAG	22	Y	Y	Y	1.812	291373	291394	Plus
34	147	1	UGGCAGUGUGGUUAGCUGGUUG	22	Y	?	Y	1.265	509536	509557	Plus
1890	123	0	UGAAAUCUUUGAUUAGGUCUGG	22	Y	?	?	1.204	1733356	1733377	Plus
988	118	24	CCCCUUGUUGCAAACCUCACGC	22	Y	?	Y	1.442	623057	623078	Minus
957	99	0	UGAAACCGUCCAAAACUGAGGC	22	Y	?	Y	1.7	464339	464360	Plus
305	96	4	AUUGUACUUCAUCAGGUGCUCUGG	24	Y	Y	Y	1.24	495601	495624	Plus
996	88	0	UGACUAGAUUACAUGCUCGUC	21	Y	Y	Y	1.437	567507	567527	Minus
87	79	0	GUGAGCAAAUUUUCAGGUGUGU	22	Y	Y	Y	1.36	1010846	1010867	Plus
12	68	3	UGAGUAUUACAUCAGGUACUGGU	23	Y	Y	Y	1.68	2720468	2720490	Minus
13	57	1	UAUCACAGCCAUUUUGACGAGUU	23	Y	Y	Y	1.268	888744	888766	Minus
92a	44	0	UAUUGCACUUGUCCCGGCC	19	Y	Y	Y	1.116	1267254	1267272	Plus
33	40	0	GUGCAUUGUAGUUGCAUUGCA	21	?	Y	Y	1.487	351656	351676	Plus
279	36	2	UGACUAGAUCCACACUCAUUAA	22	Y	Y	Y	1.437	572258	572279	Minus
79	33	6	GCUUUGGCGCUUUAGCUGUAUGA	23	Y	Y	Y	1.785	213277	213299	Plus
263	32	0	AAUGGCACUGGAAGAAUUCACGG	23	Y	Y	Y	1.981	164999	165021	Minus
7	32	0	UGGAAGACUAGUGAUUUUGUUGU	23	Y	Y	Y	1.1359	46041	46063	Plus
**2945	32	0	UGACUAGAGGCAGACUCGUUUA	22	Y	?	?	1.43	481083	481105	Plus
100-5p	30	-	AACCCGUAGAUCCGAACUUGUG	22	Y	Y	Y	1.43	1142184	1142205	Plus
100-3p	25	-	CAAGAACGGAUGUAUGGGAUUC	22	Y	Y	Y	1.43	1142224	1142245	Plus
970	21	0	UCAUAAGACACACGCGGCUAU	21	Y	?	Y	1.229	1045875	1045895	Plus
210.1	13	0	CUUGUGCGUGUGACAACGG	19	Y	Y	Y	1.512	515650	515668	Plus
999	14	0	UGUUAACUGUAAGACUGUGUCU	22	?	?	Y	1.100.	2315145	2315166	Plus
308	16	4	CGCGGUAUAUUCUUGUGGCUUG	22	Y	?	Y	1.107	508980	509001	Plus
125	7	3	UCCCUGAGACCCUAACUUGUGA	22	Y	Y	Y	1.43	1156615	1156636	Plus
210.2	6	0	UUGUGCGUGUGACAACGGCUAU	22	Y	Y	Y	1.512	515646	515667	Plus
307	6	0	CACAACCUCCUUGAGUGAGCGA	22	Y	?	Y	1.16	1859430	1859451	Minus
1000-1	6	0	AUAUUGUCCUGUCACAGCAGU	21	Y	Y	Y	1.187	325478	325498	Plus
1000-2	-	-	AUAUUGUCCUGUCACAGCAGU	21	Y	Y	Y	1.3798	224	244	Minus
375	4	0	UUUGUUCGUUUGGCUCGAGUUA	22	Y	Y	Y	1.309	1318752	1318773	Minus
309-1	4	-	UCACUGGGCAAAGUUUGUCGCA	22	Y	?	Y	1.15	907938	907959	Plus
309-2	-	-	UCACUGGGCAAAGUUUGUCGCA	22	Y	?	Y	1.602	94088	94109	Plus
932	3	-	UGCAAGCAAUGUGGAAGUGAAG	22	?	Y	Y	1.1064	154192	154213	Minus
315	2	0	UUUUGAUUGUUGCUCAGAAAGC	22	Y	Y	Y	1.612	104143	104164	Plus
927	1	0	CAAAGCGUUUGGAUUCUGAAAC	22	Y	Y	Y	1.26	2065461	2065482	Plus
**2943	1	0	UUAAGUAGGCACUUGCAGGC	20	Y	?	?	1.348	212450	212470	Plus
											
1891-1	predicted		UGAGGAGUUAAUUUGCGUGUUU	22	Y	?	?	1.199	1109750	1109771	Minus
1891-2	predicted		UGAGGAGUUAAUUUGCGUGUUU	22	Y	?	?	1.466	72802	72823	Plus
1175	predicted		AAGUGGAGUAGUGGUCUCAUCG	22	Y	?	?	1.125	1648037	1648058	Plus
1174	predicted		UCAGAUCUACUUAAUACCCAU	21	Y	?	?	1.125	1647921	1647941	Plus
993	predicted		UACCCUGUAGUUCCGGGCUUUU	22	Y	Y	Y	1.056	256798	256819	Plus
981	predicted		UUCGUUGUCGACGAAACCUGCA	22	Y	Y	Y	1.127	638380	638401	Minus
965	predicted		UAAGCGUAUAGCUUUUCCCAUU	22	Y	?	Y	1.51	2008701	2008722	Plus
316	predicted		UGUCUUUUUCCGCUUACUGCCG	22	?	Y	Y	1.289	891327	891348	Minus
285	predicted		UAGCACCAUUCGAAAUCAGUAC	22	?	?	Y	1.26	3339153	3339174	Minus
137-1	predicted		UAUUGCUUGAGAAUACACGUAG	22	Y	Y	Y	1.1191	97844	97865	Plus
137-2	predicted		UAUUGCUUGAGAAUACACGUAG	22	Y	Y	Y	1.137	260684	260705	Minus
133	predicted		UUGGUCCCCUUCAACCAGCUGU	22	Y	Y	Y	1.778	350306	350327	Minus
124	predicted		UAAGGCACGCGGUGAAUGC	19	Y	Y	Y	1.6	1664623	1664641	Minus
31	predicted		UGGCAAGAUGUUGGCAUAGCUGA	23	?	Y	Y	1.636	483257	483279	Minus
10	predicted		ACCCUGUAGAUCCGAAUUUGUU	22	Y	Y	Y	1.44	813557	813578	Plus
iab-4-1	predicted		ACGUAUACUGAAUGUAUCCUGA	22	Y	Y	Y	1.708	50265	50286	Plus
iab-4-2	predicted		ACGUAUACUGAAUGUAUCCUGA	22	Y	Y	Y	1.423	763526	763547	Minus
											
2940	125253	4125	UGGUUUAUCUUAUCUGUCGAGGC	23	?	?	?	1.222	643960	643982	Plus
2765	1162	0	UGGUAACUCCACCACCGUUGGC	22	Y	?	?	1.11	5248310	5248331	Plus
2951	1136	28	AAGAGCUCAGUACGCAGGGG	20	?	?	?	multiple			
2941-1	9	0	UAGUACGGCUAGAACUCCACGG	22	?	?	?	1.385	413147	413168	Minus
2941-2	-	-	UAGUACGGCUAGAACUCCACGG	22	?	?	?	1.385	413451	413472	Minus

aga = *An. gambiae; *ame = *Ap. mellifera; *dme = *D. melanogaster*.

Top group: miRNAs and miRNA* strands identified by deep sequencing

Middle group: predicted miRNAs identified in *Cx. quinquefasciatus *but absent from C7/10 cells were cross-referenced with the *Ae. aegypti *(AaegL1)

Bottom group: novel miRNAs identified in this study. miR-2765 (not present in miRBase v14) has recently been identified in *Bombyx mori*.

** novel miRNAs (not present in miRBase v14) recently identified in *Ae. aegypti *mosquitoes while this manuscript was under review

**Table 2:** miRNAs identified in *Cx. quinquefasciatus *adult female mosquitoes.

Culex	# miRNA	# miRNA*	Sequence	Length	aga	ame	dme	supercontig	Start	End	Strand
184	107190	0	UGGACGGAGAACUGAUAAGGGC	22	Y	Y	Y	3.567	240312	240333	Minus
317-1	71313	2	UGAACACAGCUGGUGGUAUCU	21	Y	Y	Y	3.36	1133209	1133229	Plus
317-2	-	-	UGAACACAGCUGGUGGUAUCU	21	Y	Y	Y	3.36	1134875	1134895	Plus
277	58628	0	UAAAUGCACUAUCUGGUACGAC	22	Y	Y	Y	3.36	1153785	1153806	Plus
1	36084	0	UGGAAUGUAAAGAAGUAUGGAG	22	Y	Y	Y	3.78	246250	246271	Plus
989	23667	0	UGUGAUGUGACGUAGUGGUAC	21	Y	?	Y	3.315	321364	321384	Plus
275	13910	2	UCAGGUACCUGAAGUAGCGC	20	Y	Y	Y	3.291	329815	329834	Plus
957	11682	0	UGAAACCGUCCAAAACUGAGGC	22	Y	?	Y	3.787	29593	29614	Plus
8-3p	10950	-	UAAUACUGUCAGGUAAAGAUGU	22	Y	Y	Y	3.40	815865	815886	Minus
281	9322	95	AAGAGAGCUAUCCGUCGACAGU	22	Y	Y	Y	3.64	99744	99765	Plus
Let-7	9266	5	UGAGGUAGUUGGUUGUAUAGU	21	Y	Y	Y	3.4	280610	280630	Plus
34	6301	3	UGGCAGUGUGGUUAGCUGGUU	21	Y	Y	Y	3.36	1154478	1154498	Plus
263	3749	2	AAUGGCACUGGAAGAAUUCACGG	23	Y	Y	Y	3.219	351848	351870	Minus
252-1	3157	2	(C)UAAGUACUAGUGCCGCAGGAG	21	Y	Y	Y	3.1787	6836	6856	Minus
252-2	-	-	(C)UAAGUACUAGUGCCGCAGGAG	21	Y	Y	Y	3.975	115594	115614	Plus
87	2364	0	GUGAGCAAAUUUUCAGGUGUGU	22	Y	Y	Y	3.431	379788	379809	Plus
71	2232	14	AGAAAGACAUGGGUAGUGAGAU	22	?	Y	?	3.366	117552	117573	Minus
bantam-5p	1459	-	CCGGUUUUCAUUUUCGAUCUGAC	21	Y	Y	Y	3.65	199737	199759	Minus
9	1138	440	UCUUUGGUAUUCUAGCUGUAGA	22	Y	Y	Y	3.83	64733	64754	Plus
11	888	5	CAUCACAGUCUGAGUUCUUGCU	22	Y	?	Y	3.153	639669	639690	Minus
276-1	860	2	UAGGAACUUCAUACCGUGCUCU	22	Y	Y	Y	3.136	340911	340932	Plus
276-2	-	-	UAGGAACUUCAUACCGUGCUCU	22	Y	Y	Y	3.136	541192	541213	Plus
276-3	-	-	UAGGAACUUCAUACCGUGCUCU	22	Y	Y	Y	3.2457	930	951	Plus
210.1	720	5	UUGUGCGUGUGACAACGGCUAU	22	Y	Y	Y	3.549	157657	157678	Minus
927	703	21	CAAAGCGUUUGGAUUCUGAAAC	22	Y	Y	Y	3.11	560282	560302	Plus
bantam-3p	689	-	UGAGAUCAUUUUGAAAGCUGA	21	Y	Y	Y	3.65	199698	199718	Minus
8-5p	594	-	CAUCUUACCGGGCAGCAUUAGA	22	Y	Y	Y	3.40	815904	815925	Minus
2	547	2	UAUCACAGCCAGCUUUGAAGAGC	23	Y	Y	Y	3.366	116861	116883	Minus
998	434	0	UAGCACCAUGAGAUUCAGC	19	?	?	Y	3.153	639527	639545	Minus
210.2	405	-	CUUGUGCGUGUGACAACGGCUAU	23	Y	Y	Y	3.549	157657	157679	Minus
14	358	0	UCAGUCUUUUUCUCUCUCCUAU	22	Y	Y	Y	3.676	52251	52272	Minus
285	324	5	UAGCACCAUUCGAAAUCAGUAC	22	?	?	Y	3.98	262290	262311	Minus
1890	287	0	UGAAAUCUUUGAUUAGGUCUGG	22	Y	?	?	3.64	982786	982807	Minus
190-1	231	0	AGAUAUGUUUGAUAUUCUUGGUUG	24	Y	Y	Y	3.181	347953	347976	Minus
190-2	-	-	AGAUAUGUUUGAUAUUCUUGGUUG	24	Y	Y	Y	3.351	105098	105121	Minus
283	224	0	CAAUAUCAGCUGGUAAUUCUGGG	23	Y	Y	Y	3.57	559462	559484	Plus
7	192	0	UGGAAGACUAGUGAUUUUGUUGU	23	Y	Y	Y	3.1	3357390	3357412	Minus
100	170	43	AACCCGUAGAUCCGAACUUGUG	22	Y	Y	Y	3.4	271414	271435	Plus
1891	167	1	UGAGGAGUUAAUUUGCGUGUUU	22	Y	?	?	3.829	180383	180404	Minus
999	165	0	UGUUAACUGUAAGACUGUGUCU	22	?	?	Y	3.14	96917	96938	Plus
309	33	1	UCACUGGGCAUAGUUUGUCGCAU	23	Y	?	Y	3.145	66041	66063	Minus
375	144	0	UUUGUUCGUUUGGCUCGAGUUAC	23	Y	Y	Y	3.455	42584	42605	Plus
306	143	65	UCAGGUACUGAGUGACUCUCAG	22	Y	?	Y	3.83	80436	80457	Plus
125	140	7	UCCCUGAGACCCUAACUUGUGA	22	Y	Y	Y	3.4	280975	280996	Plus
315	131	0	UUUUGAUUGUUGCUCAGAAAGC	22	Y	Y	Y	3.438	61926	61947	Plus
124	105	0	UAAGGCACGCGGUGAAUGC	19	Y	Y	Y	3.8	2074772	2074790	Plus
92b	96	0	AAUUGCACUUGUCCCGGCCUG	21	Y	Y	Y	3.722	164913	164933	Minus
1889-5p	89	-	UAAUCUCAAAUUGUAACAGUGG	22	Y	?	?	3.57	562555	562576	Plus
981-1	82	0	UUCGUUGUCGACGAAACCUGCA	22	Y	Y	Y	3.431	144482	144503	Plus
981-2	-	-	UUCGUUGUCGACGAAACCUGCA	22	Y	Y	Y	3.431	151371	151392	Plus
12	80	2	UGAGUAUUACAUCAGGUACUGGU	23	Y	Y	Y	3.57	563009	563031	Plus
31	76	2	UGGCAAGAUGUUGGCAUAGCUGA	23	?	Y	Y	3.559	256577	256599	Minus
10	59	40	CAAAUUCGGUUCUAGAGAGGUUU	23	Y	Y	Y	3.12	96000	96022	Minus
1174	58	0	CUGGGUAUUUUAGAUCAUCGGC	22	Y	?	?	3.86	865901	865922	Plus
**2945	52	0	UGACUAGAGGCAGACUCGUUU	20	Y	?	?	3.4	184461	184481	Plus
1000	49	0	AUAUUGUCCUGUCACAGCAGU	21	Y	Y	Y	3.153	102853	102873	Minus
13	37	3	UAUCACAGCCAUUUUGACGAGU	22	Y	Y	Y	3.366	116994	117015	Minus
996	36	2	UGACUAGAUUACAUGCUCGU	20	Y	Y	Y	3.19	1437010	1437029	Minus
137	33	0	UAUUGCUUGAGAAUACACGUAG	22	Y	Y	Y	3.1714	27566	27587	Minus
133	32	0	UUGGUCCCCUUCAACCAGCUGU	22	Y	Y	Y	3.1189	55748	55769	Plus
1175	35	7	AAGUGGAGUAGUGGUCUCAUCG	22	Y	?	?	3.86	866116	866137	Plus
279	26	21	UGACUAGAUCCACACUCAUUAA	22	Y	Y	Y	3.19	1441123	1441144	Minus
92a	24	-	UAUUGCACUUGUCCCGGCCUAU	22	Y	Y	Y	3.722	174912	174933	Minus
932-3p	22	-	UGCAAGCAAUGUGGAAGUGA	22	?	Y	Y	3.261	301413	301432	Minus
970	20	0	UCAUAAGACACACGCGGCUAU	21	Y	?	Y	3.495	35970	35990	Plus
316	18	0	UGUCUUUUUCCGCUUACUGCCG	22	?	Y	Y	3.496	152508	152529	Minus
305	17	1	AUUGUACUUCAUCAGGUGCUCU	22	Y	Y	Y	3.291	339134	339155	Plus
**2944a-1	13	1	GAAGGAACUUCUGCUGUGAUC	21	Y	?	?	3.66	328681	328701	Minus
**2944a-2	-	-	GAAGGAACUUCUGCUGUGAUC	21	Y	?	?	3.145	66240	66260	Minus
988	11	5	CCCUUGUUGCAAACCUCACGC	21	Y	?	Y	3.791	14331	14351	Plus
932-5p	11	-	UCAAUUCCGUAGUGCAUUGCAG	22	?	Y	Y	3.261	301450	301471	Minus
1889-3p	7	-	CACGUUACAGAUUGGGGUUUCC	22	Y	?	?	3.57	562642	562663	Plus
993	4	1	UACCCUGUAGUUCCGGGCUUUU	22	Y	Y	Y	3.12	55487	55508	Plus
278	3	0	UCGGUGGGACUUUCGUCCGUUU	22	Y	Y	Y	3.16	1026212	1026233	Plus
965	2	0	UAAGCGUAUAGCUUUUCCCAUU	22	Y	?	Y	3.48	484177	484198	Plus
Iab-4	2	1	ACGUAUACUGAAUGUAUCCUGA	22	Y	Y	Y	3.12	681163	681184	Plus
980	2	0	UAGCUGCCUAGUGAAGGGC	19	?	?	Y	3.263	352922	352940	Plus
308	3	1	CGCAGUAUAUUCUUGUGGCUUG	22	Y	?	Y	3.98	764133	764154	Plus
79	2	0	GCUUUGGCGCUUUAGCUGUAUGA	23	Y	Y	Y	3.83	80591	80613	Plus
**2943	1	0	UAAGUAGGCACUUGCAGGCAAAG	23	Y	?	?	3.121	94164	94186	Minus
**2944b-1	1	0	GAAGGAACUCCCGGUGUGAUAU	22	Y	?	?	3.66	328838	328859	Minus
**2944b-2	-	-	GAAGGAACUCCCGGUGUGAUAU	22	Y	?	?	3.145	66389	66410	Minus
											
33	predicted		GUGCAUUGUAGUUGCAUUGCA	21	?	Y	Y	3.1258	69381	69401	Minus
											
2951	162309	342	AAGAGCUCAGCACGCAGGGGUGGC	24	?	?	?	multiple			
2952	2203	-	UAGUACGGCCAUGACUGAGGGC	22	?	?	?	3.5	753922	753943	Minus
2941-1	1221	3	UAGUACGGCUAGAACUCCACGG	22	?	?	?	3.5	753643	753664	Minus
2941-2	-	1	UAGUACGGCUAGAACUCCACGG	22	?	?	?	3.5	753797	753818	Minus

aga = *An. gambiae; *ame = *Ap. mellifera; *dme = *D. melanogaster*.

Top group: miRNAs and miRNA* strands identified by deep sequencing

Middle group: predicted miR-33 was identified in C7/10 cells but absent from *Cx. quinquefasciatus*. miR-33 was cross-referenced with the *Cx. quinquefasciatus *genome.

Bottom group: novel miRNAs identified in this study.

** novel miRNAs (not present in miRBase v14) recently identified in *Ae. aegypti *mosquitoes while this manuscript was under review

miRNA expression levels, based on the number of reads obtained, varied greatly, spanning over five orders of magnitude for *Cx. quinquefasciatus *and six orders of magnitude for *Ae. albopictus *(Figure 1C, Tables 1, 2). For both species, the majority of miRNAs (>70%) were sequenced between 10 and 10,000 times (Figure 1C). miR-184 was the most highly expressed miRNA in both species, represented by 1,487,481 reads in the *Ae. albopictus *library and 107,190 reads in the *Cx. quinquefasciatus *library. In fact, miR-184 dominated the *Ae. albopictus *library, accounting for >95% of all miRNA reads. To date, miR-184 has been identified in over 39 organisms, but has no defined role in insects. Surprisingly, although small RNAs were prepared from blood-fed whole *Cx. quinquefasciatus *mosquitoes compared to *Ae. albopictus *C710 cells, these two species shared five out of the top ten most frequently occurring miRNAs: miR-184, miR-317, miR-277, miR-275, and miR-8 (Tables 1, 2). In *Drosophila*, miR-277 has predicted targets in metabolic pathways [20] while miR-8 plays a role in Wnt signaling [36]. miR-275 and miR-317 have no experimentally reported targets to date.

Mature miRNA species showed sequence lengths between 19 and 24 nt with a predominance of 22 nt and also exhibited strong bias for a 5' uracil (> 65% of all identified miRNAs) (Tables 1, 2). The presence of a 5' U is a characteristic of many miRNAs [37,38], at least in part, because strand selection of the miRNA from the miRNA:miRNA* duplex is based on the level of thermodynamic stability of the paired ends of the duplex [27,39,40].

### Mosquito miRNAs are highly conserved

The *Ae. albopictus *genome is not yet sequenced. Since miRNA sequences are highly conserved between species, we mapped miRNAs cloned from the *Ae. albopictus *cell line to the *Ae. aegypti *genome. Interestingly, all *Ae. albopictus *miRNAs and miRNA* strands mapped with 100% identity to the *Ae. aegypti *genome, indicating evolutionary constraints on not only the mature miRNA sequences, but also the pre-miRNA hairpins. 72 of the 74 *Culex *miRNA sequences mapped with 100% identity to the *Cx. quinquefasciatus *genome [35]. The identified sequence for miR-309 (Table 2) differed by one nucleotide (nt 11) from the *Cx. quinquefasciatus *genomic sequence. miR-927, occurring 700 times in the *Cx. quinquefasciatus *library (Table 2), exhibited sequence differences at nucleotides 1 and 16 compared to the *Cx. quinquefasciatus *genome. When mapped to the *Ae. aegypti *genome, one nucleotide differed from the genomic sequence. These sequence variations could not be accounted for by miRNA editing.

Several miRNA sequences mapped to multiple locations in the *Cx. quinquefasciatus *and *Ae. aegypti *genomes. Six *Cx. quinquefasciatus *miRNAs, miR-317, miR-252, miR-276, miR-190, miR-981, and miR-2944, arise from at least two possible hairpin precursors (Table 2). In *Aedes*, four miRNAs, miR-276, miR-317, miR-1000, and miR-309 arise from two potential hairpin precursors (Table 1).

With the exception of miR-33, all *Ae. albopictus *miRNAs were also identified in *Cx. quinquefasciatus *mosquitoes. Additionally, 14 miRNAs present in *Cx. quinquefasciatus *mosquitoes, but absent from *Ae. albopictus *cells, mapped with 100% sequence identity to the *Ae. aegypti *genome, and are annotated as predicted (Tables 1 and 2). Of note, *Cx. quinquefasciatus *miR-1174 was not found in *Ae. aegypti; *however, the annotated mature miRNA sequence for *An. gambiae *miR-1174 aligns to the *Ae. aegypti *genome with 95% sequence identity. Table 1 contains the predicted miR-1174 sequence for *Ae. aegypti*. Interestingly, *Cx. quinquefasciatus *miR-1174 is orthologous not to the mature miR-1174 in *An. gambiae*, but to the predicted miR-1174* (19 out of 22 nt); only these 19 nucleotides are conserved between the *Cx. quinquefasciatus *and *An. gambiae *pre-miRNAs. In total, 75 *Aedes *and *Cx. quinquefasciatus *conserved miRNAs were identified, representing over 55 seed families (Tables 1, 2).

64 of the 75 miRNAs identified in *Cx. quinquefasciatus *and *Ae. albopictus *have orthologs in *D. melanogaster*. In addition to *D. melanogaster*, we examined orthologous miRNA sequences from two other winged insects, *An. gambiae *and *Apis mellifera *(Tables 1, 2). Five miRNAs, miR-1175, miR-1174, miR-1889, miR-1890, and miR-1891, have previously been identified in *Anopheles *mosquitoes but, to date, lack orthologs in *D. melanogaster *or *A. mellifera*. Interestingly, for miR-1890, only the miRNA sequenced is conserved between *Anopheles, Culex*, and *Aedes*, and extensive sequence variations occur in the remaining arm and loop of the precursor. While this manuscript was under review, eight additional novel mosquito-specific miRNAs were identified in *Ae. aegypti *mosquitoes [41]. miR-2944a/b is present at low levels in *Cx. quinquefasciatus*; miR-2943 and miR-2945 are present at low levels in both *Cx. quinquefasciatus *mosquitoes and C710 cells (Tables 1 and 2). While orthologs of these mosquito-specific miRNAs may be identified in other organisms in the future, this group of miRNAs appears to be restricted to mosquitoes and hence, may be of more recent evolutionary origin.

### Sequence variation occurs predominantly at the 3' end of mature miRNAs

**Figure 2:**
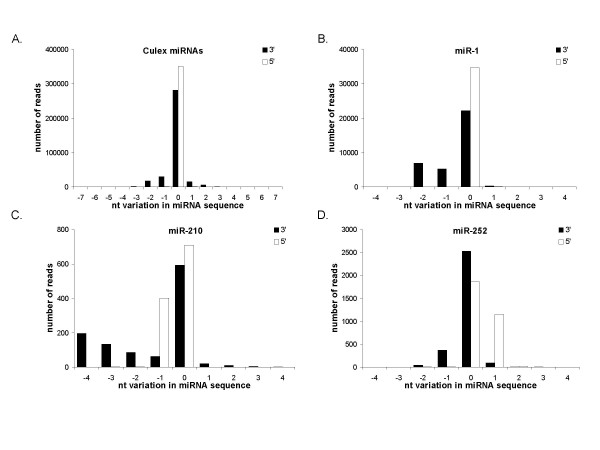
***Culex *miRNA sequence variations**. A) The total number of canonical miRNA sequence reads, annotated in Table 2, is set to "0" on the x-axis. Differences in the total numbers of canonical 3' versus 5' miRNA ends are due to greater diversity at the 3' end of a given miRNA. Negative numbers on the x-axis indicate 3' or 5' nucleotide truncations in a miRNA sequence, while positive numbers indicate 3' or 5' nucleotide additions. 58 *Culex *miRNAs (sequenced at least 10 times) are collectively represented. The numbers of reads with 3' or 5' nucleotide truncations or additions for individual miRNAs, B) miR-1, C) miR-210, and D) miR-252 are shown.

In each small RNA library, reads aligning to a given mature miRNA showed some degree of variability. Most variability occurred at the 3'ends of each mature miRNA, when compared to the 5' ends. Figure 2A depicts this variance for all conserved miRNAs present in the *Culex *library. Each canonical miRNA sequence is set at "0"; nucleotide truncations from either the 3' or 5' end are shown by negative numbers, whilst nucleotide additions are shown by positive numbers. 20.5% of miRNA reads exhibited 3' end variability compared to only 0.8% of reads for 5' variability. In accordance with other miRNA studies [18,42,43], we found that the majority of miRNAs, such as miR-1 (Figure 2B), followed this pattern of 5' sequence homogeneity and 3' heterogeneity. Precision at the mature miRNA 5' end has been reported for *Drosophila *miRNAs [44]. Such observations are congruent with the idea that the seed sequence, located within the 5' end of the miRNA, is evolutionarily constrained [15,29].

At least two miRNAs, however, did not match this trend. For both miR-210 and miR-252, two dominant miRNA species were identified (Figure 2C and 2D; Tables 1, 2). For miR-210, the most frequently occurring species was sequenced 301 times, while the second dominant species, one nucleotide longer with a cytosine at the 5' end, was sequenced 274 times. Due to variations in the 5' and 3' ends for the remaining 550 reads aligning to miR-210, the canonical 5' and 3' ends were actually represented by the second most frequently occurring sequence, which is annotated (Table 2). Interestingly, two dominant forms of miR-210, miR-210.1 and miR-210.2, one of which contains an extra 5' nucleotide, have been noted for *D. melanogaster *[18]. Furthermore, of the 19 reads aligning to miR-210 in the *Ae. albopictus *library, 13 (68%) contain an extra 5' cytosine. Only one copy of the miR-210 precursor is present in these insect genomes, therefore such differences cannot be attributed to processing from multiple pri-miRNAs. Mosquitoes and fruit flies diverged over 250 million years ago. Thus, it is striking that we see these same two forms of miR-210 expressed in mosquitoes. Our data provide strong evidence in support of the hypothesis that these two forms of miR-210 are evolutionarily conserved and are likely to function as at least partly distinct miRNAs *in vivo*.

miR-252, which maps to two loci within the *Cx. quinquefasciatus *genome, but only one locus in each of the *Ae. aegypti *and *An. gambiae *genomes, also exhibited similar variation at the 5' end (Figure 2D). The dominant, canonical miRNA species was sequenced 1,688 times, while the second dominant species, with a 5' cytosine addition, was sequenced 719 times. We also observed miR-252 variations in the *Ae. albopictus *library. 35% of the 2496 sequences aligning to *Ae. albopictus *miR-252 contained one extra 5' cytosine. The two 69 nt pri-miRNA stem-loops for *Cx. quinquefasciatus *miR-252 are 100% identical, and show 100% and 97% sequence identity with miR-252 pri-miRNA stem-loops present in the *Ae. aegypti *and *An. gambiae *genomes, respectively. Thus, these variations in the mature miRNA sequences, for both miR-252 and miR-210, do not appear to arise from differences in hairpin folding properties, and likely are a result of Drosha and/or Dicer processing.

The consequences of 5' variation can be severe, since an alteration to the 5' seed creates a new group of potential target mRNAs for a miRNA [29]. Depending on the length of the complementary seed match within a target mRNA, miRNAs arising from a single precursor, yet exhibiting 5' variation, could have both overlapping and distinct targets.

Whereas some miRNAs exhibited sequence differences at the 5' or 3' ends, we also noted differences in the ratios of miRNA:miRNA* reads when examining the *Cx. quinquefasciatus *and *Ae. albopictus *libraries. miRNA* strands for several miRNAs, including miR-8, miR-1889, and bantam, were sequenced a significant number of times, and thus are annotated with 5p or 3p (Tables 1, 2). In C7/10 cells, miR-1889-3p and miR-1889-5p were expressed at nearly identical levels, suggesting that both strands of the miRNA:miRNA* duplex are loaded equally into RISC as mature miRNAs. Interestingly, for miR-8 in the *Ae. albopictus *library, the ratio of 5p:3p miRNA reads was 1,244 miR-8-5p: 3,002 miR-8-3p (ratio of 0.41) (Table 1). In *Cx. quinquefasciatus *mosquitoes, however, the ratio was much different. miR-8-5p occurred only 594 times compared to miR-8-3p which occurred 10,950 times (ratio of 0.05) (Table 2). Of note, the dominant miRNA species for *Ae. albopictus *miR-8-3p contains one less 3' nucleotide compared to *Cx. quinquefasciatus *miR-8-3p.

We investigated the predicted miR-8 pre-miRNA structures in *Ae. aegypti, Cx. quinquefasciatus*, and *An. gambiae. Ae.aegypti *miR-8 pre-miRNA shares 98% and 92% sequence identity with the miR-8 pre-miRNA in *Cx. quinquefasciatus *and *An. gambiae*, respectively. Intriguingly, all nucleotide differences for miR-8 affect only the terminal loop of the pre-miRNA hairpin, which alters the pairing at the immediate base of the terminal loop. Thus, differences in the miRNA-5p:miRNA-3p ratios may reflect the RNA folding properties of the pre-miRNA within each species, which is known to influence strand selection. Furthermore, nucleotide diversity in the terminal loop for miR-8, a miRNA known to be involved in Wnt signaling in the fly [21,36], may help fine tune not only miRNA strand selection but also the miRNA sequence itself, thereby also fine tuning miRNA target regulation.

Whilst the total number of miRNA* strands accounted for a low percentage (<0.3%) of mapped reads in each small RNA library, some miRNA* strands were sequenced more frequently than individual miRNA species. For example, in total RNA from C7/10 cells, bantam-3p was sequenced 475 times, and therefore accounts for a greater percentage of the small RNA population than those mature miRNAs sequenced less than 400 times. Likewise, miR-281* in *Cx. quinquefasciatus *mosquitoes was sequenced 95 times, and thus accounts for a greater percentage of small RNAs than those occurring less than 95 times. Importantly, the biological relevance of the miRNA* population has been demonstrated in *Drosophila*; miRNA* strands can be loaded into Ago1-containing RISC and target complementary 3' UTRs of mRNAs [45].

### Confirmation of mosquito miRNAs

**Figure 3:**
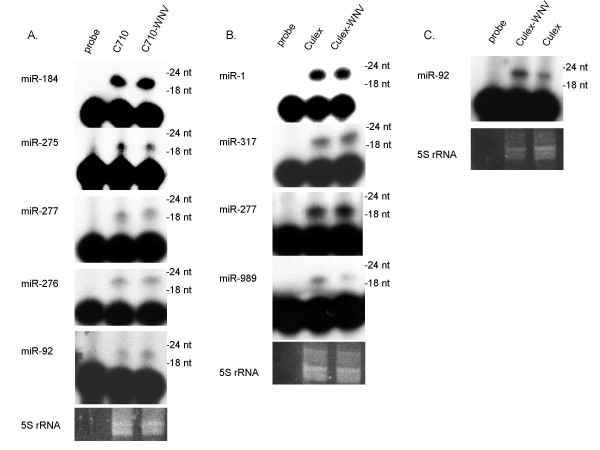
**Primer extension analysis confirms miRNA expression**. Total RNA was isolated from A) C7/10 cells or C7/10-WNV312 cells persistently infected with WNV replicons and B, C) *Cx. quinquefasciatus *mosquitoes fed a non-infectious blood meal (*Culex*) or a blood meal containing WNV-NY99 (*Culex-*WNV). 10 μg (A, C) or 4 μg (B) of RNA was used for primer extension to detect miRNAs. For each miRNA, free probe with no RNA is shown as a negative control. Ethidum bromide stained rRNA is shown as loading control.

We used primer extension analysis to confirm the expression of several of the miRNAs represented in our sequencing data. Five miRNAs, miR-184, miR-275, miR-277, miR-276, and miR-92, were sequenced >500 times and were readily detectable in total RNA isolated from C7/10 cells (Figure 3A). Five miRNAs, miR-1, miR-317, miR-277, miR-989, and miR-92 were sequenced >120 times and were readily detectable in total RNA isolated from *Cx. quinquefasciatus *mosquitoes (Figure 3B). In general, the detection level of a given miRNA reflected the overall abundance of that miRNA in the sequenced library (Figure 3, Tables 1, 2). All miRNAs analyzed by this method exhibited the expected sizes.

### Identification of novel mosquito miRNAs

To identify novel mosquito miRNAs, we used a combination of miRDeep [46] and MFold [47] to ask whether non-annotated sequences mapping to the mosquito genomes demonstrated folding properties of pre-miRNA hairpins. Each novel miRNA follows both expression and biogenesis criteria set forth for identifying new miRNAs, which include (i) a small RNA of appropriate and discrete length (19-24 nt), (ii) arising from one arm of a hairpin precursor, (iii) presence of the star strand, and (iv) evolutionary conservation [13,18,48].

**Figure 4:**
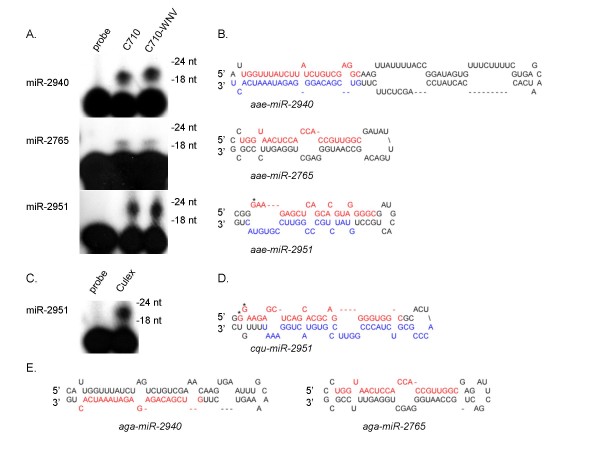
**Identification of novel mosquito miRNAs**. Primer extension analysis was used to confirm the expression of A) three novel *Ae. albopictus *miRNAs and C) one novel *Cx. quinquefasciatus *miRNAs. Total RNA was isolated from C7/10 cells or *Cx. quinquefasciatus *mosquitoes as described in Figure 3 and Methods. B) and D) Predicted pre-miRNA stem-loop structures for each novel miRNA. *Ae. albopictus *miRNAs were mapped to the *Ae. aegypti *genome, and therefore may not reflect the actual pre-miRNA structures. Mature miRNA sequences are shown in red, while corresponding miRNA* sequences identified in each library are shown in blue. For miR-2951, asterisks indicate additional 5' nucleotides present in a lower percentage of the reads mapping to each miRNA compared to the canonical sequence annotated in Tables 1 and 2. *Ae. albopictus *miR-2951 differs by one nucleotide from the *Ae. aegypti *genome. E) Pre-miRNA structures for two novel orthologous miRNAs mapping to the *A. gambiae *genome. The predicted mature miRNAs, based on sequence conservation, are shown in red.

**Figure 5:**
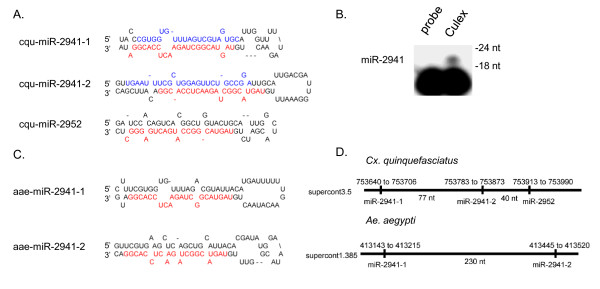
**Novel mosquito miRNAs are clustered**. A) Predicted pre-miRNA stem-loop structures for cqu-miR-2941-1, cqu-miR-2941-2, and cqu-miR-2952. B) Primer extension analysis confirms miR-2941 expression in total RNA isolated from *Cx. quinquefasciatus *adult female blood-fed mosquitoes (see Methods). C) Predicted pre-miRNA stem-loop structures for aae-miR-2941-1, and aae-miR-2941-2. As in Figure 4, mature miRNAs are shown in red, and miRNA* strands, when identified in a library, are shown in blue. D) Genomic location of the miR-2941 clusters in the *Cx. quinquefasciatus *and *Ae. aegypti *genomes. Numbers on the bottom indicate the nucleotide distance between pri-miRNA stem-loops for each miRNA.

Four new *Aedes *miRNAs (five hairpins) and three new *Cx. quinquefasciatus *miRNAs (four hairpins) were identified (Tables 1, 2). Each miRNA arises from RNA structures which fold into canonical pre-miRNA hairpins (Figures 4 and 5). Four of the new miRNAs reside on the 5p arms of their respective precursors (Figure 4B and 4C), while the remaining three miRNAs reside on the 3p arms (Figure 5). Primer extension analysis confirmed the expression of five of these miRNAs (Figures 4 and 5).

miR-2940, which lacks seed homology to any known miRNA, was amongst the most frequently recovered miRNAs present in the *Ae. albopictus *library, sequenced 125,253 times; miR-2940* was sequenced 4,125 times (Table 1). Interestingly, miR-2940 and miR-2940* are separated by 60 nt of intervening sequence, resulting in a 104 nt pre-miRNA (Figure 4B). This pre-miRNA length is unusual for metazoan pre-miRNAs, which are normally ~60 nt in length [24]. Plant pre-miRNAs, however, can be as long as 200 nt [13], and several *Drosophila *miRNAs arise from long hairpins >100 nt. The *D. melanogaster *miR-989 precursor, for example, has 99 nt of intervening sequence between the miRNA and miRNA* [18].

Two novel *Aedes *miRNAs, miR-2765 and miR-2951, arise from pre-miRNAs with typical lengths of 59 nt and 57 nt, respectively (Figure 4B). Both miRNAs were sequenced ~1,100 times; however, primer extension analysis suggested that miR-2951 is expressed at higher levels than miR-2765 (Figure 4A). Like miR-2940, miR-2765 shows no seed sequence homology to any known miRNA present in miRBase v14. miR-2951 is 100% identical to cqu-miR-2951, expressed in *Culex *mosquitoes (Figure 4C).

Whilst the majority of new miRNAs exhibited discrete lengths, as determined from both sequencing data and primer extension analysis (Figure 4 and 5), we observed variations in the 5'ends of both *Ae. albopictus *miR-2951 and *Cx. quinquefasciatus *miR-2951, which affect the seed. 29% of *Ae. albopictus *miR-2951 reads contained an additional 5' G, while *Cx. quinquefasciatus *miR-2951 reads contained 5' GG (3.4%) or 5' G (30.2%) additions or single nucleotide 5' truncations (12%) compared to the canonical sequence (54.4% of reads). Furthermore, unlike *Aedes *miR-2951*, for which a distinct sequence was identified, over five equally abundant sequences for *Cx. quinquefasciatus *miR-2951* were observed, which affect the positioning of the star strand in the pre-miRNA hairpin (Table 1, 2). Only 15 nucleotides, excluding the potential 5' seed, are conserved between *Aedes *miR-2951*, and *Cx. quinquefasciatus *miR-2951*, contributing to differences in the predicted pre-miRNA hairpin structures. These differences are also due, in part, to nucleotide differences in the terminal loops (Figure 4B and 4D). These sequence variations might also be attributed to diversity in the flanking pri-miRNA sequences; miR-2951 maps to eight locations within each of the *Ae. aegypti *and *Cx. quinquefasciatus *genomes. Notably, within each genome, all pre-miRNA loci share 100% sequence identity.

We queried three mosquito genomes (*Cx. quinquefasciatus, An. gambiae, Ae. aegypti*) present in VectorBase for the presence of each new miRNA. Both miR-2940 and miR-2765 have orthologs in *An. gambiae *(Figure 4E). The predicted precursor for miR-2765 is 93% identical at the sequence level in *An. gambiae*, while the mature miRNA sequence is 100% conserved. Interestingly, for miR-2940, the orthologous sequence mapping to *An. gambiae *chromosome X with 95% sequence identity was actually miR-2940*. Given that miR-2940* was sequenced over 4,000 times, it is possible that both strands of the miR-2940:miR-2940* duplex are loaded into RISC and function as mature miRNAs. Notably, the predicted 5p arm for *An. gambiae *miR-2940 exhibits the same seed sequence as miR-2940-5p from *Aedes*, suggesting similar functions in mRNA targeting (Figure 4B and 4E). No orthologs for miR-2951 or miR-2952 were found in *An. gambiae*. In fact, miR-2952 appears to be specific to *Cx. quinquefasciatus*.

Two additional miRNAs, aae-miR-2941 and cqu-miR-2941, are also orthologs conserved in *Aedes *and *Cx. quinquefasciatus*. cqu-miR-2941 was readily detectable by primer extension analysis in *Cx. quinquefasciatus *(Figure 5B); however, miR-2941 was sequenced only nine times in the *Ae. albopictus *library, and thus was below the limit of detection. aae-miR-2941 and cqu-miR-2941 each arise from two different pre-miRNA hairpins that map to two loci (Figure 5A and 5C). cqu-miR-2941* strands from both of the *Cx. quinquefasciatus *pre-miRNAs were identified (Table 2), indicating that both hairpins are expressed and processed. The pre-miRNAs for both aae-miR-2941 and cqu-miR-2941 are clustered within a ~350 nt stretch which, for *Cx. quinquefasciatus*, also includes another novel miRNA, miR-2952 (Figure 5D). Notably, miR-2941 and miR-2952 share the first nine 5' nucleotides and thus, have the same seed (Table 2), suggesting these two miRNAs might regulate an overlapping set of target mRNAs.

### Clusters of mosquito miRNA genes

The miR-2941 cluster represents a novel miRNA cluster present in both *Cx. quinquefasciatus *and *Aedes *mosquito genomes. To determine whether additional conserved miRNAs were clustered, we considered miRNAs which mapped to locations within 1 kb of each other. Nine mosquito miRNAs followed this pattern (Table 1, 2). The ordered distribution of each of the nine pre-miRNAs in the *Ae. aegypti *genome was similar to the distribution of pre-miRNAs in the *Cx. quinquefasciatus *genome, with two exceptions. miR-11 and miR-989 map to the plus strand in the *Ae. aegypti *genome, but map to the minus strand in *Cx. quinquefasciatus*. It is possible that this cluster is inverted in *Cx. quinquefasciatus *since (i) miR-11 and miR-989 are located on the plus strand in *An. gambiae *[35] and (ii) the order of miRNAs is still conserved. Based on sequencing reads, miRNAs within each cluster did not appear to be evenly expressed (Tables 1, 2).

### Culex miR-989 and miR-92 expression levels are altered during flavivirus infection

miRNAs are known to be important regulators of development. Additionally, miRNA expression profiles can be altered in response to environmental changes such as stress or infection. Four *An. gambiae *miRNAs, miR-34, miR-1174, miR-1175, and miR-989, show changes in expression during *Plasmodium *infection [11]. Given that *Cx. quinquefasciatus *and *Ae. albopictus *are important flavivirus vectors, we asked whether any miRNAs were aberrantly expressed during infection with WNV.

We assayed miRNA expression in WNV-replicon C7/10 cells and WNV-NY99 infected *Cx. quinquefasciatus *using primer extension. Persistent infection of C7/10 cells with WNV-replicons had no significant effect on the expression levels of the miRNAs assayed (Figures 3A and 4A). A comparison of blood-fed, uninfected female *Cx. quinquefasciatus *mosquitoes to age- and sex-matched WNV-NY99 infected mosquitoes revealed that the majority of miRNAs were unaffected; however, we observed 2.8 fold downregulation of miR-989 following WNV-NY99 infection (Figure 3B; Additional file 1, Figure S1). In contrast, miR-92 expression was upregulated in WNV-infected *Cx. quinquefasciatus *(Figure 3C; Additional file 1, Figure S1). Notably, this pattern of miRNA expression for miR-989 and miR-92 is also found in deep sequencing reads of WNV-infected *Cx. quinquefasciatus *(Additional file 2, Table S1). We also observed changes in miR-957, miR-970, miR-980, and miR-33, among others (Additional file 2, Table S1).

The targets of miR-989 and miR-92 in mosquitoes are not yet known; however, several studies have examined expression of these miRNAs during development. In *An. gambiae, An. stephensi*, and *Ae. aegypti*, miR-989 expression is restricted to female mosquitoes and found predominantly in the ovaries [10,11]. While this manuscript was in review, Li et.al. reported 454 deep sequencing of miRNAs in *Ae. aegypti *mosquitoes; miR-989 is also present in the midgut while miR-92 is present in *Ae. aegypti *embryos [41]. In the silkworm *Bombyx mori*, miR-92 is associated with embryogenesis, a stage of high cellular proliferation and differentiation [49]. Furthermore, in vertebrates, miR-92 is a member of the conserved miR-17-92 cluster and is associated with oncogenesis and increased cellular proliferation. Given the dysregulation of miR-989 and miR-92 during WNV infection, it is interesting to speculate that the targets of these miRNAs may play roles in mediating flavivirus infection in the mosquito host.

## Conclusions

This study provides experimental evidence for over 65 conserved and seven novel miRNAs present in *Aedes *and *Cx. quinquefasciatus *mosquitoes, and increases our current understanding of insect miRNAs. The majority of miRNAs identified here demonstrate conventional miRNA characteristics including evolutionary conservation, 5' end homogeneity, and an ~60 nt pre-miRNA. A small number of miRNAs were found that deviate from these standards. *Cx. quinquefasciatus *and *Aedes *miR-210, miR-252, and miR-2951 are examples of multiple, distinct miRNAs arising from one arm of a single hairpin (Figures 2 and 4). *Aedes *miR-2940, among others, arises from an unusually long pre-miRNA (Figure 4A). Additionally, the prevalence of the miRNA* strand for several miRNAs, such as miR-1889, miR-8, and bantam, expands the potential of miRNA regulation in an organism by adding to the number of possible miRNA seeds and thus adding new mRNA targets. Finally, of the novel miRNAs identified here, four currently lack orthologs in non-mosquito species, bringing the total mosquito-specific miRNAs to 16 [41].

*Aedes *and *Culex *mosquitoes are major arbovirus vectors, important in transmitting both alphaviruses and flaviviruses to humans. We found miR-989, a female-specific miRNA in *Anopheles *and *Aedes *mosquitoes, to be downregulated in WNV-infected *Cx. quinquefasciatus *while miR-92 is significantly upregulated. This is the first report of miRNA dysregulation following flavivirus infection of a natural mosquito host. Future research will elucidate the functions of these newly identified miRNAs in mosquito biology. Undoubtedly, some of the miRNAs identified here will have roles not only in mosquito development, like their *Drosophila *counterparts, but also in mediating viral infection in the mosquito host.

## Methods

### Mosquitoes and Cell Lines

*Cx. quinquefasciatus *mosquitoes (Sebring strain) were reared and maintained as previously described [50]. Female mosquitoes were fed a non-infectious blood meal containing 2 mL of Vero cells and media mixed with 2 mL of defibrinated sheep blood (Colorado Serum Company, Denver, CO) or an infectious blood meal containing 2 mL of WNV NY99 [51] infected Vero cells with media and 2 mL of sheep blood. The meals were presented separately to 200 female mosquitoes 3 to 5 day post-eclosion as previously described [50]. Mosquitoes were instantaneously killed in Eppendorf tubes by submersion in a dry ice/liquid nitrogen bath at 14 days post-blood meal and stored in RNAlater prior to RNA extraction. *Ae. albopictus *C7/10 cells were maintained at 28°C in Leibowitz L-15 media supplemented with 10% FCS, 10% tryptose phosphate broth, and antibiotics. C7/10-WNV replicon cells were generated by infecting C7/10 cells with GFP-expressing WNV replicon particles [52,53]. The cells were sorted for GFP expression 7 days post-infection, and monitored for GFP expression for one month prior to analysis to verify establishment of a persistent infection. Infection of both mosquitoes and C7/10 cells was confirmed by qRT-PCR [52].

### RNA extraction and Primer Extensions

Total RNA was prepared from ~100 whole mosquitoes and two 80% confluent T75 flasks of *Ae. albopictus *cells using TRIzol (Invitrogen) according to the manufacturer's protocol. Primer extensions were performed with 4 μg (*Cx. quinquefasciatus*) or 10 μg (C7/10) of total RNA using the AMV PE kit according to manufacturer's protocol (Promega). Oligonucleotides used for probes are listed (Additional file 3, Table S2) and were end-labeled using gamma-[32P]-ATP and T4 polynucleotide kinase. To detect individual miRNAs, a master mix was prepared for each probe and divided equally amongst the reactions. Reverse transcription products were separated on 15% TBE-urea polyacrylamide gels, exposed to film, and subjected to analysis using NIH ImageJ (Additional file 1, Figure S1).

### Small RNA cloning

Thirty micrograms of total RNA were size-fractionated on a 15% TBE-Urea polyacrylamide gel. Small RNA populations corresponding to 18-28 nt in size were extracted, eluted, and ligated to a 3' linker using T4 RNA ligase (Epicentre). 3' ligation reactions were loaded directly onto a 10% TBE-Urea polyacrylamide gel, and ligation products recovered by high-salt elution following electrophoresis. Next, a 5' linker was ligated, and products were used for SSII reverse transcription (Invitrogen). PCR reactions were carried out using the RT primer and 5' PCR primer. Linker and primer sequences are provided in Additional file 3, Table S2. Amplified cDNA products were gel-purified prior to submission for sequencing. High-throughput sequencing was performed by the Duke IGSP Sequencing Core Facility on an Illumina Genome Analyzer II.

### Bioinformatics

Sequencing reads were parsed using in-house scripts according to the following criteria: a 5' and 3' linker match of at least 4 nt and an appropriate length (18-28 nt).

To find miRNA orthologs, sequences were mapped to known miRNAs, miRNA star strands, and hairpins present in miRBase v14.0 http://microrna.sanger.ac.uk using NCBI BLAST (word size = 17, p = 85, D = 2) allowing for a 2 nt mismatch, and parsed further using Perl scripts from the miRDeep pipeline [46]. Mosquito genomes (*Cx. quinquefasciatus *Johannesburg strain and *Ae. aegypti *Liverpool strain) were obtained from http://vectorbase.org and coordinates for miRNA sequences were extracted using BLAST. For new miRNA discovery, reads mapping to each mosquito genome were analyzed using the miRDeep pipeline [46]. To further confirm novel miRNAs, reads of 19-24 nt in length occurring at least 100 times in a library were mapped to mosquito genomes, and sequences of 200 nt in length surrounding the putative miRNA were extracted, and folded using MFold [47]. FASTA files containing all unique reads for the C7/10 and *Culex *libraries as well as miRNA precursor sequences are provided (Additional files 4,5,6,7).

## Authors' contributions

RLS prepared the small RNA libraries, analyzed the data, and drafted the manuscript. DLV and SH reared the mosquitoes and performed the WNV infections. FS provided the C7/10 cells and generated the C7/10-WNV replicon cells. BRC supervised the experiments and helped draft the manuscript. All authors read and commented on the final manuscript.

## Supplementary Material

Additional file 1 **Figure S1, miRNA quantification in primer extension experiments shown in Figure **3. Primer extension experiments were quantified using NIH ImageJ. Signal ratios of (A) C7/10-WNV replicon cells: C7/10 cells and (B) WNV-infected *Culex*: uninfected *Culex *are graphed for individual miRNAs. Click here for file

Additional file 2 **Table S1, miRNA reads in *Cx. quinquefasciatus *and WNV-infected *Cx. quinquefasciatus***. Table comparing mosquito miRNA counts from high-throughput sequencing of uninfected and WNV-infected *Cx. quinquefasciatus*. The WNV-infected *Cx. quinquefasciatus *library was prepared as described in Methods. Differences in miR-989 and miR-92 expression levels are highlighted. nd = not determined Click here for file

Additional file 3 **Table S2, Oligonucleotides used in this study**. Table of oligonucleotides used for primer extension and high-throughput sequencing. Click here for file

Additional file 4 **Raw sequence data C710.fasta**. FASTA file containing sequencing reads for C7/10 *Ae. albopictus *cells Click here for file

Additional file 5 **Raw sequence data Culex.fasta**. FASTA file containing sequencing reads for *Cx. quinquefasciatus *mosquitoes Click here for file

Additional file 6 **miRNA precursor sequences Aedes_precursors.fasta**. FASTA file containing miRNA, miRNA*, and precursor sequences for *Ae. albopictus* Click here for file

Additional file 7 **miRNA precursor sequences Culex_precursors.fasta**. FASTA file containing miRNA, miRNA*, and precursor sequences for *Cx. quinquefasciatus* Click here for file

## References

[B1] Gould EA Solomon T Pathogenic flaviviruses Lancet 2008 371 500 509 10.1016/S0140-6736(08)60238-X 18262042

[B2] Mackenzie JS Gubler DJ Petersen LR Emerging flaviviruses: the spread and resurgence of Japanese encephalitis, West Nile and dengue viruses Nat Med 2004 10 S98 109 10.1038/nm1144 15577938

[B3] Gratz NG Critical review of the vector status of Aedes albopictus Med Vet Entomol 2004 18 215 227 10.1111/j.0269-283X.2004.00513.x 15347388

[B4] Ebel GD Rochlin I Longacker J Kramer LD Culex restuans (Diptera: Culicidae) relative abundance and vector competence for West Nile Virus J Med Entomol 2005 42 838 843 10.1603/0022-2585(2005)042[0838:CRDCRA]2.0.CO;2 16363169

[B5] Girard YA Klingler KA Higgs S West Nile virus dissemination and tissue tropisms in orally infected Culex pipiens quinquefasciatus Vector Borne Zoonotic Dis 2004 4 109 122 10.1089/1530366041210729 15228811

[B6] Girard YA Popov V Wen J Han V Higgs S Ultrastructural study of West Nile virus pathogenesis in Culex pipiens quinquefasciatus (Diptera: Culicidae) J Med Entomol 2005 42 429 444 10.1603/0022-2585(2005)042[0429:USOWNV]2.0.CO;2 15962797

[B7] Renault P Solet JL Sissoko D Balleydier E Larrieu S Filleul L Lassalle C Thiria J Rachou E de Valk H A major epidemic of chikungunya virus infection on Reunion Island, France, 2005-2006 Am J Trop Med Hyg 2007 77 727 731 17978079

[B8] Vazeille M Moutailler S Coudrier D Rousseaux C Khun H Huerre M Thiria J Dehecq JS Fontenille D Schuffenecker I Two Chikungunya isolates from the outbreak of La Reunion (Indian Ocean) exhibit different patterns of infection in the mosquito, Aedes albopictus PLoS One 2007 2 e1168 10.1371/journal.pone.0001168 18000540 PMC2064959

[B9] Griffiths-Jones S miRBase: the microRNA sequence database Methods Mol Biol 2006 342 129 138 16957372 10.1385/1-59745-123-1:129

[B10] Mead EA Tu Z Cloning, characterization, and expression of microRNAs from the Asian malaria mosquito, Anopheles stephensi BMC Genomics 2008 9 244 10.1186/1471-2164-9-244 18500992 PMC2430712

[B11] Winter F Edaye S Huttenhofer A Brunel C Anopheles gambiae miRNAs as actors of defence reaction against Plasmodium invasion Nucleic Acids Res 2007 35 6953 6962 10.1093/nar/gkm686 17933784 PMC2175301

[B12] Lai EC Tomancak P Williams RW Rubin GM Computational identification of Drosophila microRNA genes Genome Biol 2003 4 R42 10.1186/gb-2003-4-7-r42 12844358 PMC193629

[B13] Bartel DP MicroRNAs: genomics, biogenesis, mechanism, and function Cell 2004 116 281 297 10.1016/S0092-8674(04)00045-5 14744438

[B14] Ambros V MicroRNA pathways in flies and worms: growth, death, fat, stress, and timing Cell 2003 113 673 676 10.1016/S0092-8674(03)00428-8 12809598

[B15] Bartel DP MicroRNAs: target recognition and regulatory functions Cell 2009 136 215 233 10.1016/j.cell.2009.01.002 19167326 PMC3794896

[B16] Farh KK Grimson A Jan C Lewis BP Johnston WK Lim LP Burge CB Bartel DP The widespread impact of mammalian MicroRNAs on mRNA repression and evolution Science 2005 310 1817 1821 10.1126/science.1121158 16308420

[B17] Lagos-Quintana M Rauhut R Lendeckel W Tuschl T Identification of novel genes coding for small expressed RNAs Science 2001 294 853 858 10.1126/science.1064921 11679670

[B18] Ruby JG Stark A Johnston WK Kellis M Bartel DP Lai EC Evolution, biogenesis, expression, and target predictions of a substantially expanded set of Drosophila microRNAs Genome Res 2007 17 1850 1864 10.1101/gr.6597907 17989254 PMC2099593

[B19] Ambros V The functions of animal microRNAs Nature 2004 431 350 355 10.1038/nature02871 15372042

[B20] Stark A Brennecke J Russell RB Cohen SM Identification of Drosophila MicroRNA targets PLoS Biol 2003 1 E60 10.1371/journal.pbio.0000060 14691535 PMC270017

[B21] Brennecke J Hipfner DR Stark A Russell RB Cohen SM bantam encodes a developmentally regulated microRNA that controls cell proliferation and regulates the proapoptotic gene hid in Drosophila Cell 2003 113 25 36 10.1016/S0092-8674(03)00231-9 12679032

[B22] Leaman D Chen PY Fak J Yalcin A Pearce M Unnerstall U Marks DS Sander C Tuschl T Gaul U Antisense-mediated depletion reveals essential and specific functions of microRNAs in Drosophila development Cell 2005 121 1097 1108 10.1016/j.cell.2005.04.016 15989958

[B23] Kim VN MicroRNA biogenesis: coordinated cropping and dicing Nat Rev Mol Cell Biol 2005 6 376 385 10.1038/nrm1644 15852042

[B24] Cullen BR Transcription and processing of human microRNA precursors Mol Cell 2004 16 861 865 10.1016/j.molcel.2004.12.002 15610730

[B25] Denli AM Tops BB Plasterk RH Ketting RF Hannon GJ Processing of primary microRNAs by the Microprocessor complex Nature 2004 432 231 235 10.1038/nature03049 15531879

[B26] Forstemann K Horwich MD Wee L Tomari Y Zamore PD Drosophila microRNAs are sorted into functionally distinct argonaute complexes after production by dicer-1 Cell 2007 130 287 297 10.1016/j.cell.2007.05.056 17662943 PMC2686109

[B27] Tomari Y Du T Zamore PD Sorting of Drosophila small silencing RNAs Cell 2007 130 299 308 10.1016/j.cell.2007.05.057 17662944 PMC2841505

[B28] Campbell CL Black WCt Hess AM Foy BD Comparative genomics of small RNA regulatory pathway components in vector mosquitoes BMC Genomics 2008 9 425 10.1186/1471-2164-9-425 18801182 PMC2566310

[B29] Lewis BP Burge CB Bartel DP Conserved seed pairing, often flanked by adenosines, indicates that thousands of human genes are microRNA targets Cell 2005 120 15 20 10.1016/j.cell.2004.12.035 15652477

[B30] Brennecke J Stark A Russell RB Cohen SM Principles of microRNA-target recognition PLoS Biol 2005 3 e85 10.1371/journal.pbio.0030085 15723116 PMC1043860

[B31] Lewis BP Shih IH Jones-Rhoades MW Bartel DP Burge CB Prediction of mammalian microRNA targets Cell 2003 115 787 798 10.1016/S0092-8674(03)01018-3 14697198

[B32] Krek A Grun D Poy MN Wolf R Rosenberg L Epstein EJ MacMenamin P da Piedade I Gunsalus KC Stoffel M Rajewsky N Combinatorial microRNA target predictions Nat Genet 2005 37 495 500 10.1038/ng1536 15806104

[B33] Friedman RC Farh KK Burge CB Bartel DP Most mammalian mRNAs are conserved targets of microRNAs Genome Res 2009 19 92 105 10.1101/gr.082701.108 18955434 PMC2612969

[B34] Umbach JL Cullen BR The role of RNAi and microRNAs in animal virus replication and antiviral immunity Genes Dev 2009 23 1151 1164 10.1101/gad.1793309 19451215 PMC2763533

[B35] Lawson D Arensburger P Atkinson P Besansky NJ Bruggner RV Butler R Campbell KS Christophides GK Christley S Dialynas E VectorBase: a data resource for invertebrate vector genomics Nucleic Acids Res 2009 37 D583 587 10.1093/nar/gkn857 19028744 PMC2686483

[B36] Kennell JA Gerin I MacDougald OA Cadigan KM The microRNA miR-8 is a conserved negative regulator of Wnt signaling Proc Natl Acad Sci USA 2008 105 15417 15422 10.1073/pnas.0807763105 18824696 PMC2563117

[B37] Lim LP Glasner ME Yekta S Burge CB Bartel DP Vertebrate microRNA genes Science 2003 299 1540 10.1126/science.1080372 12624257

[B38] Du T Zamore PD microPrimer: the biogenesis and function of microRNA Development 2005 132 4645 4652 10.1242/dev.02070 16224044

[B39] Schwarz DS Hutvagner G Du T Xu Z Aronin N Zamore PD Asymmetry in the assembly of the RNAi enzyme complex Cell 2003 115 199 208 10.1016/S0092-8674(03)00759-1 14567917

[B40] Khvorova A Reynolds A Jayasena SD Functional siRNAs and miRNAs exhibit strand bias Cell 2003 115 209 216 10.1016/S0092-8674(03)00801-8 14567918

[B41] Li S Mead EA Liang S Tu Z Direct sequencing and expression analysis of a large number of miRNAs in Aedes aegypti and a multi-species survey of novel mosquito miRNAs BMC Genomics 2009 10 581 10.1186/1471-2164-10-581 19961592 PMC2797818

[B42] Lim LP Lau NC Weinstein EG Abdelhakim A Yekta S Rhoades MW Burge CB Bartel DP The microRNAs of Caenorhabditis elegans Genes Dev 2003 17 991 1008 10.1101/gad.1074403 12672692 PMC196042

[B43] Lau NC Lim LP Weinstein EG Bartel DP An abundant class of tiny RNAs with probable regulatory roles in Caenorhabditis elegans Science 2001 294 858 862 10.1126/science.1065062 11679671

[B44] Seitz H Ghildiyal M Zamore PD Argonaute loading improves the 5' precision of both MicroRNAs and their miRNA strands in flies Curr Biol 2008 18 147 151 10.1016/j.cub.2007.12.049 18207740 PMC2854039

[B45] Okamura K Phillips MD Tyler DM Duan H Chou YT Lai EC The regulatory activity of microRNA* species has substantial influence on microRNA and 3' UTR evolution Nat Struct Mol Biol 2008 15 354 363 10.1038/nsmb.1409 18376413 PMC2698667

[B46] Friedlander MR Chen W Adamidi C Maaskola J Einspanier R Knespel S Rajewsky N Discovering microRNAs from deep sequencing data using miRDeep Nat Biotechnol 2008 26 407 415 10.1038/nbt1394 18392026

[B47] Zuker M Mfold web server for nucleic acid folding and hybridization prediction Nucleic Acids Res 2003 31 3406 3415 10.1093/nar/gkg595 12824337 PMC169194

[B48] Ambros V Bartel B Bartel DP Burge CB Carrington JC Chen X Dreyfuss G Eddy SR Griffiths-Jones S Marshall M A uniform system for microRNA annotation RNA 2003 9 277 279 10.1261/rna.2183803 12592000 PMC1370393

[B49] Liu S Zhang L Li Q Zhao P Duan J Cheng D Xiang Z Xia Q MicroRNA expression profiling during the life cycle of the silkworm (Bombyx mori) BMC Genomics 2009 10 455 10.1186/1471-2164-10-455 19785751 PMC2761947

[B50] Vanlandingham DL Schneider BS Klingler K Fair J Beasley D Huang J Hamilton P Higgs S Real-time reverse transcriptase-polymerase chain reaction quantification of West Nile virus transmitted by Culex pipiens quinquefasciatus Am J Trop Med Hyg 2004 71 120 123 15238700

[B51] Beasley DW Whiteman MC Zhang S Huang CY Schneider BS Smith DR Gromowski GD Higgs S Kinney RM Barrett AD Envelope protein glycosylation status influences mouse neuroinvasion phenotype of genetic lineage 1 West Nile virus strains J Virol 2005 79 8339 8347 10.1128/JVI.79.13.8339-8347.2005 15956579 PMC1143769

[B52] Scholle F Girard YA Zhao Q Higgs S Mason PW trans-Packaged West Nile virus-like particles: infectious properties in vitro and in infected mosquito vectors J Virol 2004 78 11605 11614 10.1128/JVI.78.21.11605-11614.2004 15479801 PMC523254

[B53] Rossi SL Zhao Q O'Donnell VK Mason PW Adaptation of West Nile virus replicons to cells in culture and use of replicon-bearing cells to probe antiviral action Virology 2005 331 457 470 10.1016/j.virol.2004.10.046 15629788

