# Supplementary figures and images for: Identification of microRNAs expressed in two mosquito vectors, Aedes albopictus and Culex quinquefasciatus
Source: BMC Genomics. 2010 Feb 18;11:119. doi: 10.1186/1471-2164-11-119 (PMC2834634; doi:10.1186/1471-2164-11-119)

## Slide 1
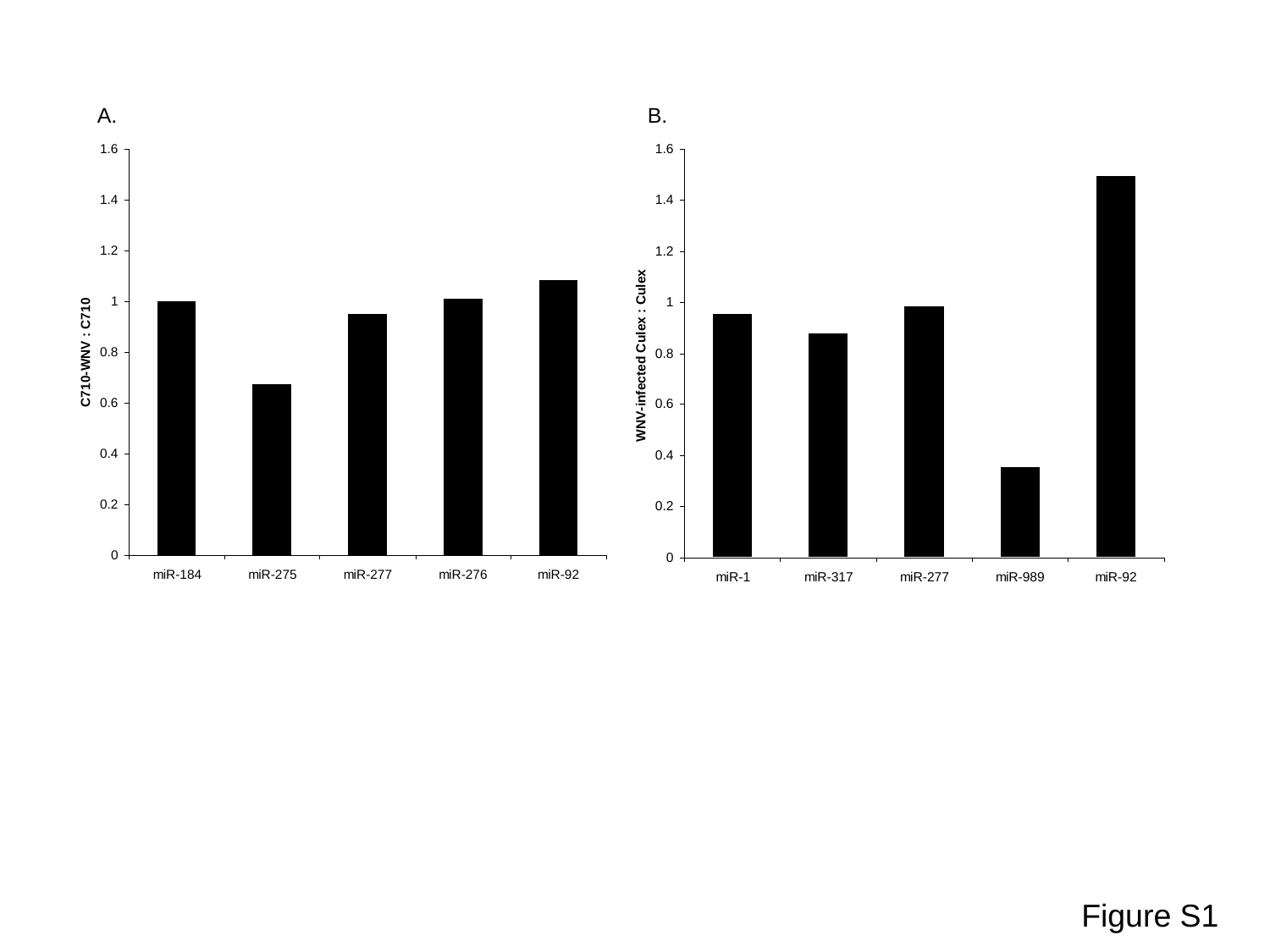

A.
B.
Figure S1

Supplement: Additional file 1 — Figure S1, miRNA quantification in primer extension experiments shown in Figure 3. Primer extension experiments were quantified using NIH ImageJ. Signal ratios of (A) C7/10-WNV replicon cells: C7/10 cells and (B) WNV-infected Culex: uninfected Culex are graphed for individual miRNAs. [file 1471-2164-11-119-S1.PPT]
