# Supplementary material for: Identification of microRNAs expressed in two mosquito vectors, Aedes albopictus and Culex quinquefasciatus
Source: BMC Genomics. 2010 Feb 18;11:119. doi: 10.1186/1471-2164-11-119 (PMC2834634; doi:10.1186/1471-2164-11-119)
Supplement: Additional file 2 — Table S1, miRNA reads in Cx. quinquefasciatus and WNV-infected Cx. quinquefasciatus. Table comparing mosquito miRNA counts from high-throughput sequencing of uninfected and WNV-infected Cx. quinquefasciatus. The WNV-infected Cx. quinquefasciatus library was prepared as described in Methods. Differences in miR-989 and miR-92 expression levels are highlighted. nd = not determined [file 1471-2164-11-119-S2.DOC]

Table S1. miRNA reads in *Cx. quinquefasciatus* and WNV-infected *Cx. quinquefasciatus*

|  | Culex | | Culex-WNV | |
| --- | --- | --- | --- | --- |
| miR- | # miRNA | # miRNA* | # miRNA | # miRNA* |
| 2951 | 162309 | 342 | 24317 | 114 |
| 184 | 107190 | 0 | 370074 | 2 |
| 317 | 71313 | 2 | 34018 | 0 |
| 277 | 58628 | 0 | 1043 | 36 |
| 1 | 36084 | 0 | 3988 | 0 |
| 989 | 23667 | 0 | 1125 | 0 |
| 275 | 13910 | 2 | 26040 | 7 |
| 957 | 11682 | 0 | 86 | - |
| 8-3p | 10950 | - | 1693 | - |
| 281 | 9322 | 95 | 364 | 9 |
| let-7 | 9266 | 5 | 826 | 0 |
| 34 | 6301 | 3 | 387 | 7 |
| 263 | 3749 | 2 | 27 | 0 |
| 252 | 3157 | 2 | 10815 | 54 |
| 87 | 2364 | 0 | 53 | 1 |
| 71 | 2232 | 14 | 31 | 7 |
| 2952 | 2203 | 0 | 487 | 0 |
| bantam-5p | 1459 | - | 27 | - |
| 2941-1 | 1221 | 3 | 139 | 0 |
| 9 | 1138 | 440 | 216 | 19 |
| 11 | 888 | 5 | 329 | 50 |
| 276 | 860 | 2 | 5405 | 5 |
| 210 | 1125 | 5 | 525 | 5 |
| 927 | 703 | 21 | nd | nd |
| bantam-3p | 689 | - | 129 | - |
| 8-5p | 594 | - | 133 | - |
| 2 | 547 | 2 | 569 | 0 |
| 998 | 434 | 0 | 133 | 0 |
| 14 | 358 | 0 | 310 | 0 |
| 285 | 324 | 5 | 1 | 0 |
| 1890 | 287 | 0 | 14 | 0 |
| 190 | 231 | 0 | 242 | 0 |
| 283 | 224 | 0 | 593 | 0 |
| 7 | 192 | 0 | 102 | 0 |
| 100 | 170 | 43 | 881 | 0 |
| 1891 | 167 | 1 | 2 | 0 |
| 999 | 165 | 0 | 48 | 0 |
| 375 | 144 | 0 | 2 | 0 |
| 306 | 143 | 65 | 627 | 0 |
| 125 | 140 | 7 | 663 | 7 |
| 315 | 131 | 0 | 0 | 0 |
| 124 | 105 | 0 | 8 | 0 |
| 92b | 96 | 0 | 21295 | 1 |
| 1889-5p | 89 | - | 288 | - |
| 981 | 82 | 0 | 5 | 0 |
| 12 | 80 | 2 | 81 | 0 |
| 31 | 76 | 2 | 39 | 0 |
| 10 | 59 | 40 | 0 | 0 |
| 1174 | 58 | 0 | 0 | 0 |
| 2945 | 52 | 0 | 112 | 0 |
| 1000 | 49 | 0 | 3 | 0 |
| 13 | 37 | 3 | 14 | 1 |
| 996 | 36 | 2 | 106 | 0 |
| 1175 | 35 | 7 | 1 | 3 |
| 309 | 33 | 1 | 0 | 0 |
| 137 | 33 | 0 | 39 | 0 |
| 133 | 32 | 0 | 1 | 0 |
| 279 | 26 | 21 | 111 | 1 |
| 92a | 24 | - | 608 | - |
| 932-3p | 22 | - | 7 | - |
| 970 | 20 | 0 | 508 | 0 |
| 316 | 18 | 0 | 1 | 0 |
| 305 | 17 | 1 | 187 | 70 |
| 2944a | 13 | 1 | 2 | 0 |
| 988 | 11 | 5 | 50 | 2 |
| 932-5p | 11 | - | 3 | - |
| 1889-3p | 7 | - | 115 | - |
| 993 | 4 | 1 | 1 | 0 |
| 278-3p | 3 | - | 7 | - |
| 308 | 3 | 1 | 7 | 0 |
| 965 | 2 | 0 | 1 | 0 |
| iab-4 | 2 | 1 | 1 | 0 |
| 980 | 2 | 0 | 267 | 0 |
| 79 | 2 | 0 | 1 | 3 |
| 2943 | 1 | 0 | 0 | 0 |
| 2944b | 1 | 0 | 0 | 0 |
| 33 | 0 | 0 | 790 | 0 |
| 278-5p | 0 | - | 96 | - |
| 2941-2 | - | 1 | - | 0 |
| Total Reads | 547802 | 1155 | 511219 | 404 |
