# Supplementary material for: Identification of microRNAs expressed in two mosquito vectors, Aedes albopictus and Culex quinquefasciatus
Source: BMC Genomics. 2010 Feb 18;11:119. doi: 10.1186/1471-2164-11-119 (PMC2834634; doi:10.1186/1471-2164-11-119)
Supplement: Additional file 3 — Table S2, Oligonucleotides used in this study. Table of oligonucleotides used for primer extension and high-throughput sequencing. [file 1471-2164-11-119-S3.DOC]

Table S2. Oligonucleotides used in this study.

| **oligonucleotide** | **Sequence (5' to 3')** |
| --- | --- |
| miR-1 probe | TCTCCATACTTCTTTACA |
| miR-92 probe | TTACAGGCCGGGACAAGTG |
| miR-184 probe | TGCCCTTATCAGTTCTCC |
| miR-275 probe | GCGCGCTACTTCAGGTAC |
| miR-276 probe | GTAGAGCACGGTATGAAGT |
| miR-277 probe | TGTCGTACCAGATAGTGC |
| miR-317 probe | TGAGATACCACCAGCTGT |
| miR-989 probe | TACCACTACGTCACA |
| miR-2940 probe | TGCCTCGACAGATAAGATA |
| miR-2765 probe | CAACGGTGGTGGAGT |
| miR-2941 probe | CGTGGAGTTCTAGCCG |
| miR-2951 probe | GCCACCCTGCGTGCTGAG |
| Sequencing 5' adaptor | GTTCArGrArGrUrArCrArGrUrCrCrGrArCrGrArCrGrArUrCrArUrGrC |
| Sequencing 3' adaptor | 5rAppTCGTATGCCGTCTTCTGCTTGT3ddC |
| RT primer | CAAGCAGAAGACGGCATACGA |
| PCR primer | AATGATACGGCGACCACCGACAGGTTCAGAGTTCTACAGTCCGA |
